# Role of HMGB1 in Cutaneous Melanoma: State of the Art

**DOI:** 10.3390/ijms23169327

**Published:** 2022-08-18

**Authors:** Federica Li Pomi, Francesco Borgia, Paolo Custurone, Mario Vaccaro, Giovanni Pioggia, Sebastiano Gangemi

**Affiliations:** 1 Department of Clinical and Experimental Medicine, Section of Dermatology, University of Messina, 98125 Messina, Italy; 2 Institute for Biomedical Research and Innovation (IRIB), National Research Council of Italy (CNR), 98164 Messina, Italy; 3 Department of Clinical and Experimental Medicine, School and Operative Unit of Allergy and Clinical Immunology, University of Messina, 98125 Messina, Italy

**Keywords:** melanoma, HMGB1, immunogenic cell death, DAMPs, biomarkers, targeted therapy, alarmins, cancer, metastasis, RAGE

## Abstract

High-mobility Group Box 1 (HMGB1) is a nuclear protein that plays a key role in acute and chronic inflammation. It has already been studied in several diseases, among them melanoma. Indeed, HMGB1 is closely associated with cell survival and proliferation and may be directly involved in tumor cell metastasis development thanks to its ability to promote cell migration. This research aims to assess the role of this molecule in the pathogenesis of human melanoma and its potential therapeutic role. The research has been conducted on the PubMed database, and the resulting articles are sorted by year of publication, showing an increasing interest in the last five years. The results showed that HMGB1 plays a crucial role in the pathogenesis of skin cancer, prognosis, and therapeutical response to therapy. Traditional therapies target this molecule indirectly, but future perspectives could include the development of new target therapy against HMGB1, thus adding a new approach to the therapy, which has often shown primary and secondary resistance. This could add a new therapy arm which has to be prolonged and specific for each patient.

## 1. Introduction

### 1.1. Cutaneous Melanoma

#### 1.1.1. Cutaneous Melanoma Clinical Types and Pathogenesis

Melanoma is considered one of the most aggressive forms of skin tumors, consisting of the abnormal growth of melanocytic cells. It has been increasing steadily as a global incidence over the past five decades [1]. Despite being a relatively rare skin cancer compared to the others (<5%), melanoma is the leading cause of skin cancer-related mortality [2]. Superficial spreading melanoma, lentigo maligna melanoma, nodular melanoma, and acral melanoma are the most common clinical types [3]. The histological classification of melanoma is useful in its diagnosis and is an important feature in defining cancer-related survival; however, its molecular subtypes are often determined by various somatic mutations [4]. Melanoma pathogenesis, defined as melanomagenesis, is based on the acquisition of sequential alterations in various genes and pathways controling metabolic or molecular mechanisms which regulate crucial cell function, survival, and replication rate [1]. When mutated, the genes involved in cancer can cause dysregulation of molecular processes with subsequent phenotypic manifestations. Main pathways leading to melanomagenesis include, but are not limited to, the mitogen-activated protein kinase (MAPK) pathway, which includes neuroblastoma RAS viral oncogene homolog (NRAS) and V-RAF murine sarcoma viral oncogene homolog B1 (BRAF), the cyclin-dependent kinase inhibitor 2A (CDKN2A) pathway, and the phosphatidylinositol-3 kinase (PI3K)/AKT/mammalian target of rapamycin (mTOR) pathway. The recognition of these mutated genes led to the development of targeted therapies, which have mostly been represented by the RAS–RAF–MEK–ERK and PI3K–AKT signaling pathways [5].

#### 1.1.2. Molecular Categories and Therapy

Skin melanoma is subdivided into three molecular categories based on gene expression: a proliferation oriented-one, driven by the SOX10–MITF pathway; an invasive one, which enhances the activity of epithelial-mesenchymal transition (EMT) and transforming growth factor-beta receptor 2 (TGFBR2); and an immune-mediated one which modifies the tumoral local environment. The three major oncogenes define three molecular forms of melanoma: BRAF-, NRAS- and KIT-mutants; a fourth subtype, also called the “triple wild-type” form, includes all the above [3]. From these data, it emerges that the molecular characterization of melanoma is necessary before the therapeutic choice, to obtain adequate clinical management and a patient-based therapy. Different molecular patterns require different types of treatment, so target therapies have been proposed over time. Target therapies are effective just with exon 15/codon 600 BRAF- mutant melanoma, adopting BRAF inhibitors in combination or not with MEK inhibitors. There is no approved drug for NRAS-mutant melanoma yet [6]. When facing triple wild-type melanoma, the only therapy option available is immunotherapy, represented by anti-programmed cell death protein 1 (PD-1) and/or anti-cytotoxic T cell antigen 4 (CTLA-4) antibodies [7]. Programmed death-ligand 1 (PD-L1) expression of tumoral cells and/or tumor-infiltrating immune cells is a pivotal requirement of anti- PD-1/PD-L1 treatment for several cancer types. However, there is no need in case of melanoma; in fact, clinical responses can be observed in a significant portion of PD-L1-negative cases [8]. Unfortunately, only adjuvant therapies are available for stages III and IV. They are represented by anti-PD-1 molecules, which are independent of mutational status, or dabrafenib and trametinib for BRAF mutant patients. The latest guidelines for the treatment of stage IV melanoma, either resected or not, state that systemic treatments are always mandatory. For first-line treatment, especially in BRAF wild-type patients, immunotherapy with PD-1 antibodies alone or in combination with CTLA-4 antibodies should be considered. In stage IV melanoma with a BRAF-V600 E/K mutation, first-line therapy with BRAF/MEK inhibitors can be proposed instead of immunotherapy [7].

### 1.2. HMGB1

#### 1.2.1. Structure and Localization

High mobility group box 1 (HMGB1) is a nuclear protein, encoded by a gene on chromosome 13q12, that acts as a non-histone chromatin-binding protein, with 215 amino acids and a molecular weight of 25-kDa. HMGB1 is composed of two proximal homologous DNA binding domains and a C–terminal ending with residues of glutamic and aspartic acid [9]. HMGB1 is normally localized in the nucleus, but it can move to the cytosol, or it can be released or actively secreted in the extracellular space by injured cells. Inside the nucleus, HMGB1 binds chromatin, stabilizes DNA, and regulates gene transcription by binding/bending duplex DNA. After damage signals, it shuttles from the nucleus to the cytoplasm or is released, thus affecting multiple cell stress responses and inflammatory mechanisms [10]. The translocation of HMGB1 from the nucleus to the cytoplasm is driven by the nuclear localization signals (NLS1 and NLS2) and nuclear export signals (NESs). Exogenous stimuli, which cause injury or death, lead to cellular alarm system activation thus releasing alarmins or pathogen-associated molecular patterns (DAMPs) to signal danger-induced cellular stress [9,10].

#### 1.2.2. Mechanism of Release and Receptors

Two HMGB1 release mechanisms are known: passive release and active secretion. HMGB1 is passively released from damaged or necrotic cells triggering an immediate inflammatory response via pro-inflammatory cytokines such as TNFα. Active secretion of HMGB1 occurs via immune cells, endothelial cells, platelets, neurons, astrocytes, and tumor cells during stress or secondary to other DAMP signals as reinforcement [11].

HMGB1 can bind to several extracellular receptors, such as the one for advanced glycation end products (RAGE), Toll-like receptor (TLR) 9, TLR4, TLR2, integrins, mucin domain (TIM-3), and C-X-C chemokine receptor type 4 (CXCR4) [9]. Given the plethora of possible target receptors for HMGB1, each domain can interact with different molecules and, in particular, residues 150–183 bind to RAGE, a major actor in the beginning and maintenance of the inflammatory process. RAGE, belonging to the immunoglobulin superfamily, is a transmembrane receptor with an extracellular domain, a transmembrane domain, and a cytoplasmic tail of 43 amino acids. While the extracellular domain is responsible for ligand binding, the cytoplasmic tail is involved in intracellular signal transduction [2]. Firstly, described as a receptor for advanced glycation end products (AGE), RAGE is now recognized as a multi-ligand receptor, including HMGB1. RAGE is involved in HMGB1-induced cell inflammation, proliferation, migration, and immunity. Furthermore, extracellular HMGB1 stimulates RAGE expression in several cytotypes [12].

#### 1.2.3. Function

HMGB1 plays a key role in both acute and chronic inflammation. In physiological conditions, this protein is inside the nucleus of quiescent macrophages/monocytes. Cell stress or death are the main mechanisms that lead to the release of HMGB1 outside the cell membrane, thus HMGB1 functions as an alarmin, meaning a molecule that triggers an inflammatory response in combination with other cytokines, DAMPs, and pathogen-associated molecular patterns (PAMPs) [13]. HMGB1 plays a central role in autophagy induction, an endogenous survival mechanism against cell stress [14]. HMGB1 induces autophagy in the nucleus upregulating the expression of heat shock protein (HSP) 27, while in the extracellular space, once released by cancer cells, it binds RAGE inducing in turn autophagocytic activity in nearby cells [9]. It is demonstrated that cancer can upregulate autophagy, which leads to drug resistance thwarting chemotherapy [11]. HMGB1-induced autophagy can also be regulated by several miRNAs, a family of small non-coding RNAs which, through epigenetic mechanisms, play an important role in the maintenance of immune homeostasis, cancer progression, and inflammation. Recently, it was demonstrated that several miRNAs modulate HMGB1 expression and its functions [9]. Moreover, miRNAs can affect HMGB1 gene expression, modulating cancer progression. HMGB1 is a direct target of miR-548b, and the expression level of this miRNA can suppress melanoma cells’ growth by targeting the HMGB1 pathway [15].

This narrative review aims to assess the role of this molecule in the pathogenesis of human melanoma and its potential therapeutic role. This review has been conducted by researching the PubMed database, and the results are sorted by year of publication.

## 2. Discussion

### 2.1. HMGB1-Related Melanoma Growth

**Figure 1:**
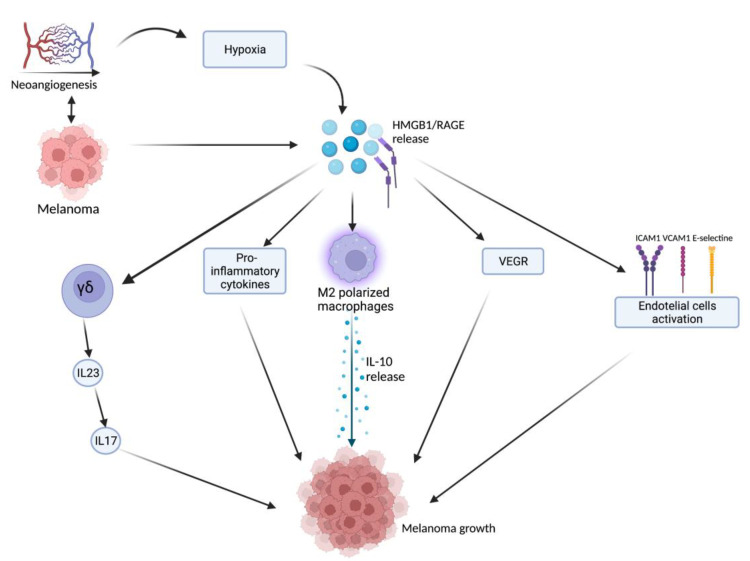
Tumor microenvironment and hypoxia, caused by cancer cells’ metabolic and oxygen demands, enhance HMGB1 release. HMGB1 binds to RAGE and actives several pathways: γδ-T cells, pro-inflammatory cytokines release, M2-macrophages, VEGF activation, and endothelial cell activation. All these lead to melanoma growth through the maintenance of an inflammatory microenvironment. Created with BioRender.com.

HMGB1, after being released from damaged or necrotic cells, is used by the immune system to recognize tissue damage in order to initiate repair responses and to promote lymphocyte maturation [16]. From here, we can deduce a role in oncological diseases [16]. One of the important stages of disease progression is neoangiogenesis, which is fundamental for sustaining the metabolic and oxygen demands of cancer cells. It is known that solid tumors exhibit large hypoxic areas because of an imbalance between their oxygen supply and consumption. This hypoxic environment results in focal areas of tumor cell necrosis with consequent release of DAMPs and alarmins, including HMGB1 [17]. Extracellular HMGB1, released from the tumor cells under hypoxia, mediates communication between cells in the tumor microenvironment through binding several receptors, especially RAGE and TLR4, which contribute to tumor growth via sustenance of long-term inflammation [16]. Once secreted by melanoma cells, HMGB1 binds to RAGE, activating the endothelial cells with consequent increased expression of the adhesion molecules VCAM-1, ICAM-1, and E-selectin [16]. Within the tumor necrotic zones, the release of HMGB1 leads to the activation of kappa-light-chain-enhancer of activated B cells (NF-κB), which in turn upregulates leukocyte adhesion molecules and the production of pro-inflammatory cytokines and angiogenic factors, including vascular endothelial growth factor (VEGF) [16]. Tumor-Associated Macrophages (TAMs) are recruited in the tumoral milieu characterized by the presence of chemokines and proinflammatory factors [18,19,20]. After tumor infiltration, macrophages can acquire two different phenotypes: the M1 phenotype exerts a cytotoxic effect on tumor cells, with increasing production of nitric oxide (NO) and reactive oxygen species (ROS), which mediate the apoptosis of neoplastic cells [21]. Tumor cells create a microenvironment that promotes the acquisition of an M2 phenotype, which possesses pro-tumor characteristics, and, in contrast to M1 phenotype, exhibits low cytotoxic properties with defective production of NO and ROS, promoting the growth and vascularization of tumor cells [21,22]. HMGB1, released by melanoma cells, promotes the accumulation of M2-macrophages, which enrich the tumoral microenvironment with IL-10, turning down the tumoral killing [22]. Further evidence that the production of HMGB1/RAGE-dependent IL-10 by macrophages promotes melanoma growth has come from a human melanoma tissue study, in which high infiltration of IL-10-producing macrophages was detected in melanoma tissue with a high expression of HMGB1 [17]. Since HMGB1 directly induces IL-10 production in TAMs, blocking IL-10 with a neutralizing antibody led to delayed tumor growth in a B16 mouse melanoma model [17]. RAGE/HMGB1 axis also activates T lymphocytes, as demonstrated by a study with animal specimens: the study showed that the HMGB1/RAGE axis influences melanoma growth via the expression of IL-23 and IL-17 from a subpopulation of T cells, (γδ-T cells) [23]. Growth of melanoma cell line B16-F10 was significantly inhibited, and expression of IL-23 and IL-17 was markedly reduced in RAGE−/− mice compared with wild-type mice. The same study also showed that HMGB1 stimulates the production of IL-23 in a RAGE-dependent manner, which in turn promotes the expression of IL-17; subsequently, IL-17 promotes tumor growth through IL-6 induction with the consequent activation of the Signal transducer and activator of transcription 3 (STAT3) [23]. In addition to promoting carcinogenesis, interleukin production, and shift of T cell subpopulation, the HMGB1/RAGE axis has been shown to play a key role in suppressing cytotoxic T cell activity by increasing PD-L1 expression levels [24,25]. Moreover, HMGB1 levels were higher in patients who did not respond to the immune checkpoint inhibitor ipilimumab than in responding patients, supporting the hypothesis that the HMGB1/RAGE axis also leads to a tumor-promoting microenvironment [26]. In addition, RAGE was detected in the cytoplasm of human melanoma cells (G361 and A375) and the treatment with AGEs induced the proliferation and migration of human melanoma cells. Thus, treatment with anti-RAGE antibodies could explain the inhibition of tumor formation, invasion, and increase in the survival rate of specimens in an in vivo animal model [27]. AGEs and RAGE could be a valuable target in the near future for the treatment of melanoma since their levels are abundantly inferior in healthy skin, thus suggesting that this type of therapy could have potentially very few side effects [28]. The main pathways involved in melanoma growth are represented in Figure 1.

### 2.2. HMGB1 and UVB

Among the risk factors for melanoma, exposure to ultraviolet (UV) rays is certainly the best known. By suppressing skin immunity, UV rays ease the initiation of skin lesions and establish tumoral evasion mechanisms. In the setting of UVB-induced DNA damage, a time-dependent increase in the release of damage-associated molecular patterns such as HMGB1 has been detected [25,29]. The expression of PD-L1, an immune checkpoint molecule, which can inhibit effector T cell activity and reduce anti-tumor immunity, was shown to significantly increase in melanoma cells after UV exposure. HMGB1, secreted by melanocytes and keratinocytes after UVR irradiation, binds to RAGE thus promoting the downstream NF-κB- and interferon regulatory factor 3 (IRF3) -dependent transcription of PD-L1 in melanocytes, furthering survival mechanisms. UV exposure significantly reduced the susceptibility of melanoma cells to CD8+ T cell-dependent cytotoxicity through activation of the HMGB1/TBK1/IRF3/NF-κB cascade which in turn triggers the PD-1/PD-L1 checkpoint. We can deduce that the increased levels of PD-L1–UV lead to the suppression of immunity in the cutaneous microenvironment, promoting immune evasion of cancer cells and inducing the onset and progression of melanoma [25]. Targeting the UVB-induced HMGB1/RAGE axis could inhibit PD-L1 induction in UVB-exposed melanocytes and melanoma cells, which may serve as potential drug targets to mitigate immune escape of malignant and premalignant melanocytes. This could play a role both in the therapeutic setting, treating patients with marked photodamage [25]. Furthermore, another key mechanism linked to acute and chronic UV exposure is the TLR4-dependent inflammatory dysregulation. HMGB1 secreted by keratinocytes in response to UV induces the activation of TLR4 signaling which enhances the migration of melanoma cells, furthering the hypothesis that TLR4 plays a pivotal role in UV-driven progression and metastasis of this tumor, thus explaining one of the main features of melanoma, which is linfovascular metastatization [30]. TLR4 was found to be most expressed in melanoma tissue. MiR-145-5p, a TLR4-expression antagonist that inhibits carcinogenesis and metastasis via the NF-κB signaling pathway, is downregulated in tumor tissue of patients with melanoma [30]. Targeting the TLR4-signaling pathway has shown promising results in preclinical and clinical investigations using small molecule modulators of natural and synthetic origin [30]. Moreover, the expression of HMGB1 receptor RAGE, in its surface form, increases over time with a positive trend even after a single UVB dose, suggesting a positive feedback mechanism that could sustain the HMGB1 production. Finally, lower levels of RAGE act upon UVB-induced resistance to apoptosis and response to UV damage, with overall increased tumor resistance to oxidative damage and subsequent cellular death and may have implications for early stages of melanoma development or as a predictor of disease progression [29].

### 2.3. HMGB1 as a Marker

Currently, no consensus on the use of blood tests for monitoring melanoma recurrence exists. A plethora of molecules have been evaluated for their potential clinical values as melanoma biomarkers, such as lactate dehydrogenase (LDH), tyrosinase, and PD-L1 [2]. Nevertheless, despite progress in the prevention and early detection of melanoma, biomarkers available to date present several limitations and, for this reason, there is currently no ideal biomarker for melanoma. Among the molecules studied as possible future markers of disease activity, one is represented by RAGE, a receptor for HMGB1, which perpetrates the local inflammatory levels and was evaluated as a marker of disease activity. Since RAGE levels are increased in the environment surrounding melanoma cells and cancer cells themselves, is safe to assume that targeted therapies against RAGE signaling may represent a new strategy, although further studies are needed to make any sensible statements [27]. Increasing attention is being paid to several of its ligands, including HMGB1, as a sophisticated signal of danger with a pleiotropic function, which can serve as a possible biomarker of disease and prognostic marker of therapeutic response. On this topic, Li et al. demonstrated that HMGB1 levels were overexpressed in melanoma samples when compared to normal skin and nevi tissues. It was also noted that higher levels of HMGB1 correlate with more severe disease stages and with worse survival rates in melanoma patients [31]. Interestingly, elevated levels of HMGB1 positively correlated with several clinicopathological features of melanoma, among them tumor thickness, mitotic index, and metastases [31]. To further explore the possible role of HMGB1 as a prognostic marker, the connection between this molecule and the status of melanoma cell proliferation was studied by measuring the mitotic index. Higher HMGB1 levels showed a positive correlation with mitotic index, which in turn is linked to advanced stages of melanoma [31,32]. Wang et al. demonstrated the dual role of HMGB1 in cancer [33]. Excessive production of HMGB1 causes chronic inflammatory responses, mediated by the release of cytokines such as IL-6 and IL-8 which, in turn, stimulate carcinogenesis through tumor cell proliferation, angiogenesis, EMT, invasion, and metastatization [33]. On the other hand, nuclear HMGB1 plays a protective role in tumor suppression and tumor chemoradiotherapy and immunotherapy, reducing potential side effects occurring after systemic therapies that target not only tumoral cells but also actively replicating, healthy cells, such as the bone marrow, and basal layer-based cells [33]. Nucleus-located HMGB1 promotes the regulation of telomeres and the maintenance of genome stability [9]. Therefore, the roles of HMGB1 in the regulation of DNA damage repair and carcinogenesis suggest that targeting HMGB1 could provide a new therapeutic perspective, opening a possible new line of research [33]. The role of HMGB1 has been evaluated also in association with other molecules, such as interferon-inducible protein 1 (IRGM). Tian et al. investigated the function of IRGM in human melanoma demonstrating that overexpression of IRGM was related to melanomagenesis. By blocking the translocation of HMGB1 from the nucleus to the cytoplasm, IRGM1-mediated cellular autophagy is inhibited, thereby reducing cell survival. This evidence confirmed that IRGM is an independent risk factor that promotes melanoma progression and is associated with poor patient survival. It’s safe to assume that IRGM may be a prognostic marker as much as a therapeutic target [34].

Despite the immunogenicity demonstrated in several studies, malignant melanoma is characterized by rapid progression and primary or secondary resistance to treatment. Ipilimumab (Ipi), a monoclonal antibody against the human CTLA-4, has proven to be one of the most effective immunotherapy drugs for melanoma therapy, with a clinical response rate of only 10%. A study conducted to evaluate the differences in the sera between responder and non-responder patients demonstrated an early increase in eosinophil counts as well as a decrease in S100A8/A9 and HMGB1 in responding melanoma patients. Conversely, higher baseline neutrophil and monocyte counts, as well as serum levels of S100A8/A9 and HMGB1, indicated a lack of response to Ipi therapy. This data represents further evidence of the possible use of HMGB1 both as a prognostic marker and as a marker to predict the therapeutic response [26]. HMGB1 was also investigated as a biomarker response for Boron neutron capture therapy (BNCT), a non-invasive therapeutic technique for treating malignant tumors but, as a newly developed technique, results are too scarce to make any final considerations [35]. Beyond the aforementioned ones, recent studies have also evaluated the role of another RAGE mediator, namely S100B, as a possible biomarker. S100B, a small EF-hand calcium-binding protein in the intracellular space interacts with the transcription factor p53 inhibiting its transcriptional activity, thus resulting in a decrease in p53-dependent apoptosis and a consequent increase in melanoma cell survival. S100B is used as a prognostic factor and predictor of overall survival [2,36]. S100B was found to play a role as a prognostic biomarker of treatment response: when secreted by tumors, higher levels of S100B are predictive of poorer outcomes [37]. Moreover, it is used in the management of melanoma to predict response to therapy [37,38]. Therefore, from our research, we can affirm that there is an urgent need to identify suitable biomarkers to improve early diagnosis, precise staging, and prognosis, but most of all therapy selection and monitoring biomarkers are needed to select the appropriate therapy and follow-up the patient with a non-invasive method.

### 2.4. Melanoma Metastases and HMGB-1-Based Possible Future Therapies

HMGB1 is closely associated with cell survival and proliferation and may be directly involved in tumor cell metastasis development thanks to its ability to promote cell migration, enhance the adhesive properties of cells, and rearrange components of the extracellular matrix [9]. Serum HMGB1 interacts with the cell-surface receptor RAGE, which is a primary signaling pathway triggering the onset of various diseases and, most importantly, in the maintenance of chronic inflammation. HMGB1 binds to RAGE, which then activates several signaling molecules including NF-κB extracellular signal-regulated kinase (ERK1/2) and p38. HMGB1 can bind also to TLR2 and TLR4, which, through Myeloid differentiation primary response 88 (MyD88), activate the expression and release of pro-inflammatory cytokines, such as TNF and IL-6 [2]. HMGB1 also activates the NF-κB pathway through interaction with CXCL12/CXCR4, thus inducing the chemotaxis and recruitment of inflammatory cells [39]. HMGB1 combined with TIM-3 induces the secretion of VEGF, promoting tumor angiogenesis, which is the first step toward the metastasic process [39]. Among the inflammatory cells that play a role in the metastasis process, M2 polarized TAMs can help the tumor to overcome a hypoxic environment in order to support its progression. Hypoxia-induced HMGB1 attacks M2-TAMs, which secrete IL-10. IL-10, via regulatory T cells, suppresses CD8+ T lymphocytes. Moreover, IL-10 induces the downregulation of molecules involved in antigen presentation to CD8+ T lymphocytes, thus promoting immunoregulatory responses, inducing T cell regulation and suppression of the production of pro-inflammatory cytokines [40]. Given these premises, it can be hypothesized that inhibiting HMGB1 activity during treatment may positively affect antitumor therapy. It has been hypothesized that HMGB1 knockout in melanoma cells may suppress tumor growth in vivo via CD8+ T cells and accelerate the infiltration of CD8+ T cells, macrophages, and the activation of dendritic cells resident in tumor tissues. On this topic, a study conducted by Yakomizo et al., showed that the knockout of HMGB1 in tumor cells converted tumors from scarcely immunogenic phenotypes to inflammation-prone-ones, de facto inhibiting in vivo tumor growth. Thus, manipulation of tumor derived HMGB1 might be applicable to improve the clinical outcome of cancer therapies, including immune checkpoint blockades and cancer vaccine therapies [41]. Highly metastatic tumor cells preferentially enter senescence and adopt survival mechanisms, while apoptosis predominates in weakly metastatic tumor cells. This has been seen to be related to HMGB1 levels, suggesting that HMGB1 modulation in tumors with different metastatic states could be useful in disease containment. However, advanced stages of metastasis may represent a limitation to this strategy [42]. Recently, agents targeting the MEK–ERK1/2 pathway or immune checkpoints have emerged as an effective treatment to improve progression-free survival and overall survival for patients with stage III and stage IV melanomas. It must be pointed out, though, that they have limitations since in the former, resistance to therapy is obtained within 13 months, while in the latter reverse dysfunctional antitumor T-cell states and induced durable antitumor responses occur in a relevant percentage of patients [43]. However, BRAFi + MEKi induce lasting regression of melanoma through immune-mediated mechanisms. The BRAFi + MEKi treatment promotes the cleavage of gasdermin E (GSDME) and the consequent release of HMGB1, a marker of pyroptotic cell death. Unfortunately, BRAFi + MEKi-resistant melanoma cells lack pyroptosis markers, thus dampening the original inflammatory processes, and show decreased intratumoral T-cell infiltration but are still sensitive to pyroptosis-inducing chemotherapy. These data implicate that BRAFi + MEKi-induced pyroptosis in antitumor immune responses is a valid therapeutic strategy and highlights possible new therapeutic approaches for resistant melanoma [43].

A growing interest is emerging in the Role of miRNAs: small single-stranded non-coding RNA molecules, as therapeutic agents which can stop the progression of malignancies by the reintroduction of miRNAs into a population of cancer cells or by using the mRNA antagonist. One of the most relevant miRNAs in melanoma, MiR-548b, was significantly downregulated in tumor samples when compared to adjacent normal tissues and relates to worse overall survival in patients with melanoma. Overexpression of miR-548b suppresses the growth and metastasis-linked traits of melanoma cells. HMGB1 is a target of miR-548b, and its expression level is negatively regulated by miR-548b, while the reintroduction of HMGB1 abolishes the inhibiting effects of miR-548b on melanoma cells. All these findings demonstrated that miR-548b might act as a cancer-suppressive miRNA in human melanoma by inhibiting HMGB1, thus suggesting potential systemic and local usefulness [15]. On a side note, miRNAs have also been evaluated for their role in drug response modulation, in particular for dabrafenib. Namely, miR-26a is involved in the upregulation of dabrafenib efficacy via an HMGB1-dependent autophagy pathway in melanoma. The treatment with a miR-26a mimic and HMGB1 shRNA increased the efficacy of dabrafenib in melanoma cells, according to one study [44]. These results shed light on a novel treatment for conventional dabrafenib-based chemotherapy for melanoma and its potential mechanism.

**Figure 2:**
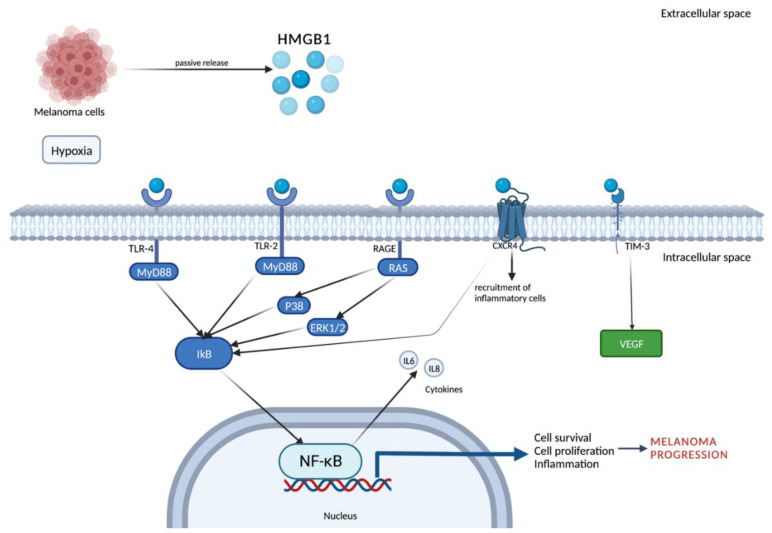
HMGB1 is released into the extracellular environment through a passive mechanism by melanoma cells. HMGB1 binds to RAGE, TLR2, and TLR4 and transduces cellular signals through a common pathway that induces the NF-κB pathway. The activated NF-κB translocates to the nucleus. HMGB1 also interacts with CXCR4 to activate the NF-κB pathway and induce chemotaxis and recruitment of inflammatory cells. The interaction of HMGB1 and TIM-3 induces the secretion of VEGF to promote tumor angiogenesis. All these pathways promote cell survival, cell proliferation and, finally, melanoma progression. Created with BioRender.com.

Switching to peptides, they play a relevant role in cell biology and many diseases including cancer. Peptide Rb4, derived from protein proteolipid protein 2 (PLP2), acts directly on tumor cell multiplication inducing the expression of two DAMPs molecules, HMGB1 and calreticulin, which trigger immunoprotective effects in vivo against melanoma cells. Overexpression of PLP2 increased tumor metastasis while the suppression of PLP2 inhibited the growth and metastasis of melanoma cells. This evidence may suggest that peptide Rb4 could act as a promising adjuvant to be developed as an anticancer drug [45]. Finally, other compounds such as glycyrrhizin have been studied as coadjuvants in melanoma therapy. It has been reported that this product inhibits pulmonary metastasis in mice inoculated with B16 melanoma (a murine tumor cell line used for research as a model for human skin cancers) by regulating the HMGB1/RAGE and HMGB1/TLR4 signal transduction pathways. The inhibition of the HMGB1/RAGE pathway reduces NF-κB expression, phosphorylation, and nuclear translocation which altogether induces cellular invasion. Moreover, RAGE/NF-κB signaling stimulates TGF-beta secretion which, in turn, increases the process of migration and invasiveness, thus starting the development of metastasis [46]. Aloin (ALO) has also been studied as the major anthraquinone glycoside extracted from the Aloe species whose anti-tumoral effects are well known but not fully understood. ALO was demonstrated to exert protective effects in melanoma-promoting cell apoptosis via the inhibition of HMGB1 release in melanoma cells. HMGB1 was demonstrated to facilitate ALO-mediated apoptosis by binding to its receptor, TLR4, and activating extracellular regulated protein kinases (ERK) signal pathways. Although ALO cannot be suggested to eradicate melanoma, this remedy may be combined with the more conventional cytotoxic chemotherapy or any other methods to interfere with cancer progression [47]. The main findings about the role of HMGB1 in melanoma metastasis and possible future therapies are summarized in Table 1. The molecules and pathways involved in melanoma metastatization are represented in Figure 2.

### 2.5. HMGB1 and Immunological Cell Death

As already stated, DAMPs are molecules that once secreted, released, or exposed to the surface by dying or injured cells, secrete adjuvant or dangerous signals for the immune system. Among the DAMPs, the main actors are surface-exposed calreticulin (CRT), secreted adenosine triphosphate (ATP) and passively released HMGB1: they represent the main hallmarks of immunogenic cell death (ICD) of cancer cells [48]. Although extracellular HMGB1 is essential for the development of ICD-mediated immunogenicity, it is also associated with tumor progression. In recent years, ICD has emerged as a possible therapeutic approach for the development of novel therapeutics for the treatment of tumors, in which cytotoxic compounds promote both cancer cell death and the release of DAMP from dying cells [49]. These DAMP molecules recruit and activate dendritic cells (DCs) that present tumor-specific antigens to T cells that clear out the neoplastic cells. The ability of some anticancer therapies to induce ICDs depends on their ability to induce endoplasmic reticulum (ER) stress and reactive oxygen species (ROS) production, both of which are essential components that activate danger-associated intracellular signaling pathways [50]. Interestingly, the expression of DAMP molecules occurs in a stress-dependent manner in the ER. To date, only a limited number of chemotherapy drugs can activate the ICD of cancer cells, which may be classified into two groups: group I ICD inducers target DNA and repair machinery proteins, cytosolic proteins, plasma membrane, or nucleic proteins; examples are chemotherapeutic agents including anthracyclines, oxaliplatin (OXP), and ultraviolet C irradiation. The endoplasmic reticulum is the target of group II ICD inducers, which include photodynamic therapy and Coxsackievirus B3 [51].

Imiquimod (IMQ) is a synthetic ligand of toll-like receptor 7 that exerts antitumor activity and is already topically used in non-melanoma skin cancer and lentigo maligna melanoma in hard-to-treat areas, such as the face. IMQ stimulates cell-mediated immunity or directly induces apoptosis. In a mouse model of B16F10 melanoma, IMQ reduced tumor growth, either by direct injection in situ or by vaccinating mice with IMQ. IMQ-related tumor-specific T cell proliferation promoted tumor-specific cytotoxic killing by CD8 + T lymphocytes, thus increasing the infiltration of immune cells into the tumor [52]. In a study conducted by Giglio et al., mitoxantrone and doxorubicin, two pro-ICD agents, stimulated the release of high levels of HMGB1 in melanoma cell lines, thus confirming that both agents could induce cell death. HMGB1 was released passively in the extracellular space by dying cells both in wild-type and BRAF mutated melanoma cells [53].

Also, the RT53 peptide was demonstrated to mediate anticancer effects by selectively inducing cancer cell death in vitro and in vivo in cells treated with this peptide. Plasma membrane exposure of CRT, the release of ATP, and the exodus of HMGB1 from dying cancer cells through membranolytic action were detected [54].

Switching to photodynamic therapy (PDT), it is a minimally invasive anti-cancer treatment widely used in clinical practice. It usually uses 5-aminolaevulinic acid (ALA) or its methylated ester (MAL), two light-responsive prodrugs, which, after irradiation with a red light of ~630nm, stimulate cytotoxic ROS causing tumor-selective destruction and providing low side effects [55]. PDT-induced oxidative stress can also trigger immune responses against cancer cells through the induction of ICD [56]. In a mouse model, PDT treatment with ML19B01 and ML19B02, two structurally related ruthenium photosensitizers, induced death of melanoma cells containing hallmarks of the ICD including HMGB1, which in turn activated antigen-presenting cells resulting in efficient phagocytosis by dendritic cells [57]. Another protocol investigated the PDT effects using different concentrations of aluminum-phthalocyanine (AlPcNE) in mice models. The exposure of DAMPs, namely HMGB1, CRT, and ATP, all hallmarks of ICD, and the presence of apoptotic and necrotic cells were assessed. PDT induced ICD in B16F10 cells proportionally to the concentration of AlPcNE [58]. This evidence suggests the potential role of PDT in inducing ICD, even using different types of photosensitizers. PDT as an ICD-inducing approach can be particularly interesting for the in situ immunotherapy of superficial tumors, such as melanoma. This local approach is further justified by the relatively low availability of treatment modalities for this cancer type.

Moving to systemic therapies, radiotherapy and chemotherapy are standard cancer therapies, although cancer cells often develop resistance to these treatments [59]. Hyperthermia, through radiosensitization, reduces the resistance of cancer cells inducing enhanced immune responses. The association of hyperthermia plus radiotherapy induces ICD in irradiated melanoma B16F10 cells, with an ICD-related mechanism. Indeed, HSP70, HMGB1/DNA complexes and ATP, markers of ICD, were detected. Chronic inflammation is linked to tumorigenesis and extracellular HMGB1 behaves like a pro-inflammatory cytokine that induces the expression of further inflammatory factors, favoring the persistence of the inflammation mechanism which in turn manipulates the immune system [60].

Immunepotent CRP (ICRP) is a mixture of substances that were shown to have cytotoxic activity on different tumor cell lines in vitro and can modulate the immune response. ICRP was demonstrated to be cytotoxic in B16F10 melanoma cells, increasing the rate of cell death when combined with Oxaliplatin (OXP). When administered alone, OXP treatment only induced CRT exposure, ATP, and HMGB1 release, while the combination of ICRP + OXP managed to increase the levels of DAMPs, exposure of CRT, and release of ATP, HSP70, HSP90, and HMGB. It can be suggested that ICRP may enhance the release of ICD-molecules with antitumor abilities which can prevent and block the growth of melanoma like an antitumor drug [61].

**Figure 3:**
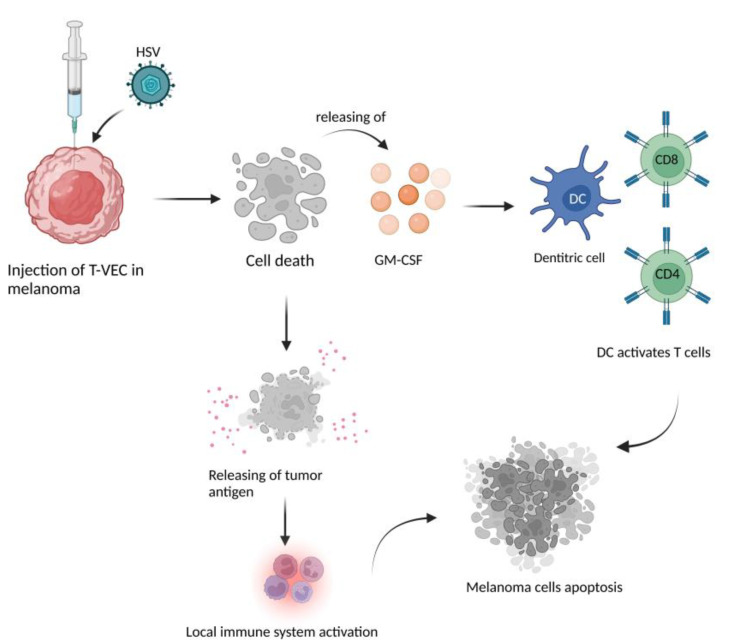
T-VEC is subcutaneously injected directly into the site of cancer. T-VEc attacks the melanoma cells locally, but also triggers the release of GM-CSF which recruits and actives dendritic cells. Dendritic cells activate CD8+ and CD4+ cells which, in turn, attack melanoma causing cell apoptosis and tumor reduction. Created with BioRender.com.

In recent years, increasing scientific attention is being paid to the role of oncolytic viruses both as a single therapy and as a combined therapy for the treatment of unresectable melanoma representing a leading search field. Studies have shown that the mechanism through which these viruses act is the ICD model, through the release of mediators such as HMGB1, CRT, HSP70, HSP90, and ATP. Oncolytic viruses have emerged as promising vectors for treating cancer which can selectively enter, replicate, and lyse tumor cells [62]. Currently, only one oncolytic virus has been approved by the Food and Drug Administration for the treatment of melanoma, the Talimogene Laherparepvec (T-VEC), a herpes simplex virus 1 (HSV-1). It is a gene-modified variant obtained through deletion of γ34.5 and ICP47 and insertion of GM-CSF to enhance therapeutic activity and attenuate pathogenicity [63]. Phase I, II, and III clinical trials were concluded with promising results from the use of T-VEC in the treatment of melanoma [64,65,66]. T-VEC contacts tumor cells directly entering the tumor environment, normally by local injection, and starting replication which leads to consequent lysis of the infected tumor cell and release of tumor antigens, thus stimulating the local immune response [67]. In addition, the GM-CSF expression stimulates migration and maturation of dendritic cells and subsequent antigen presentation to CD4+ and CD8+ T cells, which reach distant metastases [63]. Figure 3 summarizes the mechanism through which T-VEC acts in melanoma.

A 2017 randomized, open-label phase 2 trial compared Ipi (*n* = 100) with the T-VEC plus Ipi (*n* = 98) in the treatment of 198 patients with unresectable, stage IIIB–IV melanoma. In the combination group, 38 (39%) of 98 patients achieved an objective response, the primary endpoint, to treatment versus 18 (18%) of 100 in the Ipi group. T-VEC plus Ipi showed an almost doubled response rate and a higher degree of regression in visceral metastases with no unexpected increase in toxicity. This study suggested that oncolytic viruses and checkpoint inhibitors appear to have a more favorable therapeutic window than other immunotherapy combinations [68]. Another study sampled twenty-nine clinical strains of HSV-1 to identify the strain with the most potent oncolytic ability in order to further optimize HSV-1-based immunotherapy. Viral strains have been reworked through deletion of the genes encoding ICP34.5 and ICP47, and the insertion of a gene encoding a form of the envelope glycoprotein of gibbon ape leukemia virus (GALV-GP-R-) which provides tumor selectivity and enhance the immunogenicity of cell death. The expression of GALV-GP-R- improved the oncolytic ability in several tumor cell lines in vitro and mouse xenograft models. The increased immunogenic cell death in vitro was confirmed by the release of HMGB1 and ATP and by high levels of CRT on the cell surface. This new HSV-1-based platform may allow the development of new oncolytic immunotherapies; these are believed to be more effective in combination with other anticancer agents, in particular the blockade of the immune checkpoint targeting PD1/PDL1 [69]. Additionally, HF10, a spontaneously mutated strain of HSV-1 with a deletion mutation in some viral genes, was used in an in vitro study revealing relevant cytolytic effects in murine and human melanoma tumor cells injected with the virus [70].

In addition to HSVs, other oncolytic viruses are also currently being studied. Among these are Coxsackievirus A21 [71] and Oncolytic Newcastle disease virus (NDV) [72]. A study aimed to investigate whether the Oncolytic NDV strain, NDV/FMW, induces ICD in melanoma cells by assaying the expression and release of ICD markers in melanoma cell-derived tumors in intratumorally injected mice. CRT exposure, the release of HMGB1 and HSP70, HSP90, and the secretion of ATP in melanoma cells were detected, thus suggesting that oncolytic NDV/FMW might be a potent inducer of ICD in melanoma cells, which cooperates with several other forms of cell death [72]. As already mentioned, the main therapeutic concern of melanoma is the primary and secondary resistance to therapy connected to the immune evasion mechanisms of the tumor. To improve the clinical conditions of patients, stimulation of the host’s immune system or direct lysis of abnormal cells represents a valid therapeutic option in the future, as genetic engineering techniques are improving the safety and efficiency of the vector. Furthermore, the most recent studies are showing that it is possible to associate oncolytic viruses with other therapies already on the market, with promising therapeutic results. The main findings about the role of HMGB1 in the development of ICD in melanoma and its possible use as a novel therapeutic approach are summarized in Table 2.

## 3. Conclusions and Future Perspectives

HMGB1 is a molecule that originates from damaged cells. In physiological conditions, this protein is located inside the nucleus, but cell stress leads to its release outside the plasma membrane, either by passive release or active secretion. The main function of HMGB1 is that of an alarmin which binds several extracellular receptors, such as RAGE, TLR9, TLR4, TLR2 integrins, which subsequently trigger an inflammatory response. Among the stimuli that cause overexpression of HMGB1 it’s worth mentioning several types of cancers, including melanoma. The collective amount of works presented in this study suggests that HMGB1 plays a role in carcinogenesis, both via the inflammatory stimulus and the modification of tumoral microenvironment. Elevated HMGB1 serum levels have been detected in patients with melanoma. Extracellular HMGB1 behaves as a paracrine/autocrine factor for cancer growth, proliferation, migration, and angiogenesis, as high levels of HMGB1 positively correlate with metastasis development risk. Furthermore, HMGB1 is closely associated with disease severity and poor prognosis, thus validating the role of this molecule both as a diagnostic and prognostic marker. Endogenous HMGB1 can serve as a marker to predict the therapeutic response, as higher baseline levels have been shown to predict failure or poor response to systemic therapies. Beyond the utility of a new biomarker for disease management, HMGB1 might, in the future, help to select patient-specific therapies and evaluate therapy responsiveness. The therapeutic potential of HMGB1 has been confirmed in several in vivo and in vitro studies. Targeting extracellular HMGB1 induces positive effects in cancer management by reducing inflammatory tissue damage and causing a thorough remodeling of the immunologic actors involved in it. Among the molecules capable of targeting HMGB1, miRNAs might exert cancer-suppressive effects, suggesting their potential systemic and local usefulness. Several miRNAs, acting alone or in combination with chemotherapy, can modulate and attenuate HMGB1 expression, thus inhibiting inflammation and reducing tumor growth and metastatic processes. Further investigation and studies on HMGB1/miRNA correlation may provide a new line of research for a novel therapeutic approach. Another option is represented by anti-HMGB1 monoclonal antibodies, as more and more biologic therapies are used in every field, not least in the management of melanoma. To date, there are no available molecules that specifically target HMGB1 for the treatment of human melanoma, however, studies on animal subjects are underway for the therapy of several inflammatory diseases: in the near future, dampening the inflammatory processes in the case of skin tumors as well might represent an effective strategy for growth inhibition and metastasis risk. Finally, HMGB1 plays a role in the ICD, representing its main hallmark. Various therapies act via an ICD-induced mechanism, as in the case of PDT or chemotherapy. To this day, the most interesting field of research is represented by oncolytic viruses, either as a single therapy or combined to already known medications for unresectable melanomas. All these findings open new perspectives for the development of cancer therapies, aimed to improve patients’ prognosis and overall survival. It is becoming increasingly clear that there cannot be a single cure for all patients with melanoma. We are entering an era in which the chemotherapeutic approach is being surpassed, as side effects leave us longing for more tailored and precise approaches to each patient. In fact, both treatment personalization and combinatorial targeting of different immune defects represent the way forward to cure a disease that just a few years ago was considered incurable, let alone in the ending stages of its natural course. Further efforts in the study of HMGB1 in pathologies with an increasingly high incidence, such as melanoma, are necessary to reach the distribution of anti-HMGB1 drugs.

## Figures and Tables

**Table 1:** The role of HMGB1 in metastasis process and possible future therapies.

Topic	Author, Reference	Study Characteristics
Metastases	Yokomizo, K et al. [41]	In a mouse model, knockout of HMGB1 in tumor cells converted tumors from scarcely immunogenic phenotypes to inflammation-prone-ones, inhibiting in vivo tumor progression.
Metastases	Lee, Y-Y et al. [42]	HMGB1 plays a role in the senescence or apoptosis of cancer cells. Highly metastatic tumor cells preferentially enter senescence and adopt survival mechanisms, while apoptosis predominates in weakly metastatic tumor cells.
Possible future therapies	Yu, Y et al. [44]	miR-26a is involved in the upregulation of dabrafenib efficacy via an HMGB1-dependent autophagy pathway in melanoma. The treatment with a miR-26a mimic and HMGB1 shRNA increased the efficacy of dabrafenib in melanoma cells.
Possible future therapies	Maia, V.S.C. et al. [45]	Peptide Rb4 acts directly on tumor cells inducing the expression of HMGB1, which trigger the immunoprotective effect in vivo against melanoma cells.
Possible future therapies	Hiramoto, K et al. [46]	Glycirrizin inhibits HMGB1/RAGE pathway and reduces NF-κB expression, phosphorylation and nuclear translocation, which altogether induces cellular invasion.
Possible future therapies	Li, P et al. [47]	HMGB1 facilitates ALO-mediated apoptosis by binding to its receptor, TLR4, and activation of ERK signal pathways.

**Table 2:** The main findings about the role of HMGB1 in the development of ICD in melanoma and its possible use as a novel therapeutic approach.

Topic	Author, Reference	Study Characteristics
ICD	Huang, SW et al. [52]	Exposure of HMGB1 and calreticulin in a mouse model of B16F10 melanoma, following direct injection in situ or vaccinating mice with IMQ, reduced tumor growth, demonstrating the role of HMGB1 in ICD.
ICD	Giglio, P et al. [53]	Mitoxantrone and doxorubicin could induce cell death through the release of high levels of HMGB1 in melanoma cell lines.
ICD	Konda, P et al. [57]	In a mouse model, PDT treatment with ML19B01 and ML19B02 induced dying melanoma cells contained hallmarks of the ICD, including HMGB1.
ICD	Chesney, J et al. [68]	T-VEC plus Ipi showed an almost doubled response rate and a higher degree of regression in visceral metastases with no unexpected increase in toxicity. The ICD was confirmed by the release of HMGB1.
ICD	IGNYTE study [69]	The expression of GALV-GP-R- improved the oncolytic ability in several tumor cell lines in vitro and mouse xenograft models. The increased immunogenic cell death in vitro was confirmed by the release of HMGB1 and ATP and by high levels of CRT on the cell surface.
ICD	Shao, X et al. [72]	HMGB1, HSP70, HSP90, CRT exposure and the secretion of ATP in melanoma cells were detected in melanoma cells line after treating with oncolytc NDV/FMW, demonstrating the role of these molecules in the ICD.

## Data Availability

Not applicable.
